# Real-time bacterial microcolony counting using on-chip microscopy

**DOI:** 10.1038/srep21473

**Published:** 2016-02-23

**Authors:** Jae Hee Jung, Jung Eun Lee

**Affiliations:** 1 Department of Electrical Engineering, California Institute of Technology, Pasadena, CA 91125, USA; 2 Center for Environment, Health, and Welfare Research, Korea Institute of Science and Technology, Seoul 136-791, Republic of Korea; 3 Han-River Environment Research Center, National Institute of Environmental Research (NIER), Yangpyeong-gun, Gyeonggi-do 476-823, Republic of Korea

## Abstract

Observing microbial colonies is the standard method for determining the microbe titer and investigating the behaviors of microbes. Here, we report an automated, real-time bacterial microcolony-counting system implemented on a wide field-of-view (FOV), on-chip microscopy platform, termed ePetri. Using sub-pixel sweeping microscopy (SPSM) with a super-resolution algorithm, this system offers the ability to dynamically track individual bacterial microcolonies over a wide FOV of 5.7 mm × 4.3 mm without requiring a moving stage or lens. As a demonstration, we obtained high-resolution time-series images of *S. epidermidis* at 20-min intervals. We implemented an image-processing algorithm to analyze the spatiotemporal distribution of microcolonies, the development of which could be observed from a single bacterial cell. Test bacterial colonies with a minimum diameter of 20 μm could be enumerated within 6 h. We showed that our approach not only provides results that are comparable to conventional colony-counting assays but also can be used to monitor the dynamics of colony formation and growth. This microcolony-counting system using on-chip microscopy represents a new platform that substantially reduces the detection time for bacterial colony counting. It uses chip-scale image acquisition and is a simple and compact solution for the automation of colony-counting assays and microbe behavior analysis with applications in antibacterial drug discovery.

Microbiological research techniques often rely on the accurate determination of the number of colony forming units (CFUs). Bacterial growth is an essential indicator for the selection of antibiotics^1^, toxicology tests^2^, and the evaluation of food and drug safety^3^. Counting visible microbial colonies (which can be sampled from various sources, such as water, air, and soil) grown on semi-solid agar-based growth media is the conventional method for quantitative microbiological analysis of a broad spectrum of prokaryotic and eukaryotic microbes. Major advantages of colony-counting assays include the simple protocols and high sensitivity for detecting growing cells (i.e., a single culturable cell in a sample can develop into a visible colony). However, since the development of colony-counting assays a century ago, the technique has changed little. Microbial colonies are still grown in conventional Petri dishes or multi-well plates. Visual plate counting is commonly implemented using aliquots of liquid cultures and plating out of serial dilutions onto culture plates. Following incubation under conditions appropriate for the microorganism of choice, the colonies are counted to determine the number of CFUs. This is done manually by counting colonies on plates illuminated using transmitted light, which is a time-consuming process that is vulnerable to human error. Furthermore, microbial colony-counting method requires relatively long culturing times to enable the microbes to multiply sufficiently to form visible colonies. For clinical applications, for example, long analysis times for slow-growing microbial strains can delay the initiation of appropriate antimicrobial medical therapy. Moreover, long analysis times incur excessive costs in pharmaceutical and healthcare product manufacturing applications.

The need for faster microbial enumeration has driven the development of automated bio-imaging technologies. To eliminate the manual counting of colonies, image processing techniques have been developed to automate colony-counting systems. Such systems have used digital cameras or scanners to image the cell colonies in agar media in Petri dishes, where the colonies were enumerated using an image processing algorithm^4^,^5^,^6^,^7^,^8^. Several automated colony-counting systems are commercially available, including the ProtoCOL automated counters and the Whitley aCOLyte (Synbiosis, Cambridge, UK). These algorithms consist of the following image processing steps: 1) elimination of the rims of Petri dishes; 2) identification of threshold values to isolate colonies from the background; 3) dividing colonies using segmentation techniques such as the distance transform^9^, Hough transform^10^, watershed transform^11^, or fuzzy logic^12^; and 4) counting the colonies using the compactness ratio to remove noise. Such automation systems may also integrate motion control for translating the sample substrate or digital camera^13^.

These automation procedures eliminate the tedious manual counting process and reduce the scope for human error. However, although these techniques can be applied for purposes of high-throughput colony counting, the conventional colony culturing of microbes still requires relatively long times to reach the detectible colony sizes, which makes the colony counting processing slow. To overcome this problem, Frost developed a method for rapidly detecting microbial growth using microscopic detection of nascent microcolonies^14^. The use of microscopy can deliver enumeration results substantially faster than the standard plate counting methods. However, with standard microscopy, the resolution is inversely proportional to the field-of-view (FOV) of the image; therefore, observation of the entire culture area with high resolution requires several images. Moreover, the culture plates must be removed from the incubator for observation, which is inconvenient and may disturb the colony, resulting in long intervals between observations. Therefore, to cover large areas for measuring microcolonies with high resolution and, hence, reducing the detectible colony size, automated motion control is required to translate either the objective lens or the sample. London *et al*. (2010) reported a fully automated colony-counting platform called the Growth Direct^TM^ System, which consisted of an incubator, robotic moving parts to translate the sample, and a digital imaging system. This system was capable of measuring cellular autofluorescence to detect and enumerate the microcolonies^15^. However, the fully automated colony-counting systems were designed primarily for quality control in the food production and pharmaceutical industries, which require detection of a large number of samples of many different bacterial species; they are not widely used in research laboratories due to their high cost^16^,^17^.

Lensless on-chip imaging strategies not only reduce the size and the cost of the systems but also bypass the inherent limitations of lenses, such as optical aberrations and the trade-off between the FOV and the resolution. In recent years, two main streams of lensless on-chip microscopy techniques have been extensively reported: (i) diffraction-based digital in-line holography^18^,^19^,^20^,^21^,^22^,^23^,^24^ and (ii) contact-mode shadow imaging-based microscopy^25^,^26^,^27^,^28^,^29^.

The first group of lensless on-chip microscopes relies on computation to render images of target objects from interferometry measurements of the objects’ scattered light field. In a typical digital in-line holographic microscopy setup, a coherent light source in combination with a small pin-hole, is used to create a spherical wave that illuminates the object. The interference pattern between the unscattered reference wave and the wave scattered by the sample is used to reconstruct the wavefront at the object plane. As a result of the development of high-resolution digital image sensors, digital hologram recording and the numerical reconstruction method are now available, new tools for biological microscopy that simplify the conventional microscopes and overcome the limitation of lenses^30^,^31^.

In the second group of lensless on-chip microscopes, the sample is placed in contact with the pixel array of the image sensor. When illuminated, the direct shadow created by the sample can be collected at the photodiode of each pixel in the image sensor^32^,^33^. In this scheme, the imaging resolution is limited to the twice the pixel size of the sensor as defined by the Nyquist theorem. Additionally, the image quality is dependent on the contrast of the sample and the distance between the sample and the detector^34^. As one branch of shadow imaging, optofluidic microscopy (OFM) used the motion of the specimens in the microfluidic channel which is translated over an aperture array on an image sensor^25^,^35^. OFM improves spatial resolution by exploiting the time domain in the acquisition process. The optical resolution is determined by the size of the apertures fabricated on the image sensor pixels, and the most sharpness is achieved at the floor of the microfluidic channel^25^,^36^. Another type of contact-mode shadow-imaging technique involves sub-pixel sweeping microscopy (SPSM)^27^. (Details are provided in following section). The shifting of the illumination source can be used to digitally control the movements of object shadows on the sensor array as a function of the source position and can also lead to the generation of a higher-resolution shadow image.

Here, we describe a novel microcolony-counting system based on on-chip microscopy. This system is capable of automated acquisition of time-series images of bacterial colonies as well as real-time enumeration and sizing during continuous incubation. Integration of the culture agar media into a complementary metal–oxide–semiconductor (CMOS) sensor enables real-time image generation of the entire culture area. Specifically, the built-in imaging capability allows for fast and easy tracking of the microcolony growth without requiring external microscopy. Our system uses a low-cost, lensless on-chip microscopy imaging using a CMOS image sensor as an alternative to conventional microscopy. We believe that this represents the first demonstration of on-chip microscopy for rapid automated microcolony counting. For the on-chip microscopy platform, we used our recently developed ePetri system with a SPSM technique. This system features a high spatial resolution (HR) of 660 nm, with bright-field imaging over a wide FOV (i.e., 5.7 mm × 4.3 mm) using a low-cost, compact configuration^27^,^29^,^37^. With SPSM, a moving light source creates multiple shadowed images of a sample located directly on the image sensor. These low-resolution (LR) shadow images are then processed using a super-resolution algorithm to provide the HR images^27^. In our previous work, the microscope used for SPSM was an automated self-imaging Petri dish platform (ePetri) consisting of a CMOS image sensor, light-emitting diode (LED) array for illumination, and a thermoelectric cooler (TEC)^29^. The system was capable of automated imaging with a subcellular resolution over the full FOV of the entire CMOS area.

In this study, we describe the implementation and operation of a real-time microcolony-counting system based on the ePetri on-chip microscopy platform. A few microliters of the bacterial suspension are dropped onto the CMOS chip directly, and it is covered with a small piece of nutrient agarose sheet. The device then monitors bacterial colony growth in real time. We obtained micron-scale bacterial colony size distributions, with a time for colony enumeration of only 6 h for *S. epidermidis*. The performance of the system was compared with verified manual counts. Our results indicate that this system is capable of rapid and robust real-time microcolony monitoring for biomedical and bioscientific applications.

## Results

### System setup

**Figure 1:**
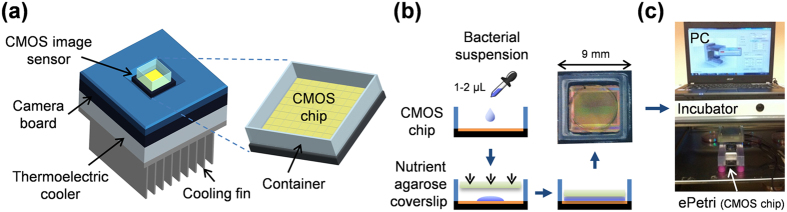
Schematic diagram showing the real-time bacterial microcolony counter system using on-chip microscopy. (**a**) The ePetri platform consisted of a CMOS image sensor chip, a camera board, and a thermoelectric cooler (TEC) with a cooling fin. (The entire ePetri platform with the LED array is shown in Fig. S1). The polymethylmethacrylate (PMMA) container part (10 mm × 10 mm × 3 mm) was bonded to the CMOS image sensor using polydimethylsiloxane (PDMS). The CMOS image sensor chip was mounted onto the camera board via a customized sensor socket, which allows easy maintenance and replacement of the sensor chip. (**b**) The preparation procedure of the real-time measurement of bacterial microcolonies consisted of only two steps: loading the bacterial suspension onto the CMOS chip, and covering the CMOS chip with the nutrient agarose sheet. (**c**) After these preparation steps, the ePetri platform was placed inside an incubator to commence the acquisition of time-series images of the bacterial microcolonies.

Figure 1(a) shows a schematic diagram of the ePetri platform, which consists of a CMOS sensor chip, a camera board, and a TEC. (The ePetri platform with LED array is shown in more detail in Fig. S1). The CMOS image sensor has an imaging area of 5.7 mm × 4.3 mm and is composed of 2.2-μm pixels (Aptina MT9P031). A 10 mm × 10-mm polymethylmethacrylate (PMMA) container was bonded onto the CMOS image sensor. The sensor chip was mounted onto the camera board via a customized sensor socket for signal readout. One side of the TEC was attached to the socket to protect the bacterial cells from the heat generated by the sensor circuit, and the other side was cooled using a fan and plate-fin heat sink, which was a standard component for cooling a central processing unit (CPU).

We grew *Staphylococcus epidermidis* bacterial colonies on the CMOS image sensor. As shown in Fig. 1(b), following injection of ~1 μL of the bacterial suspension on the sensor, it was covered with a thin agarose sheet with nutrient broth (Becton Dickinson, Franklin Lakes, NJ), which served to grow bacteria. The entire CMOS chip, including the container, was sealed using a transparent polydimethylsiloxane (PDMS) cover to prevent desiccation of the agarose sheet^38^,^39^,^40^. When the bacterial cell was on the surface of the CMOS image sensor, it could directly record a shadow image of the sample. The passive layers between the sample and sensor (i.e., sensor glass cover and the micro-lens layer) were removed to prevent deterioration of the imaging resolution due to diffraction of the sample shadows (further details can be found in the Method section)^29^. The resolution was limited to approximately twice the pixel size, based on the Nyquist criterion. To improve the pixel-limited resolution, we applied an SPSM super-resolution algorithm^41^,^42^. Briefly, we placed the sample above the image sensor surface, tilted the illumination angle to induce a sub-pixel shift of the sample shadow over the image sensor, and then captured a series of low-resolution (LR) images for each angle. We then calculated the shift based on the height of the sample above the pixels. We interpolated the LR images onto a larger matrix according to the corresponding shifts and reconstructed a high-resolution (HR) image (see Fig. S1).

During the image acquisition process, the system was placed inside a standard 37 °C incubator (see Fig. 1(c)). A laptop running a custom MATLAB program was used to control the LED array, the TEC, and the fan as well as the CMOS image sensor to collect images at 20-min intervals. Following image acquisition, another custom MATLAB super-resolution program was used to process the LR images and produce an HR image for each imaging interval.

### Microcolony imaging

Time-series HR image sequences of a growing bacterial colony were compared with the corresponding LR raw images captured directly by the sensor (see Fig. S2). The raw LR images were used to construct HR images using SPSM with a shift-and-add pixel super-resolution algorithm. From the reconstructed HR image, the boundary of the bacterial colony was clearly resolved.

**Figure 2:**
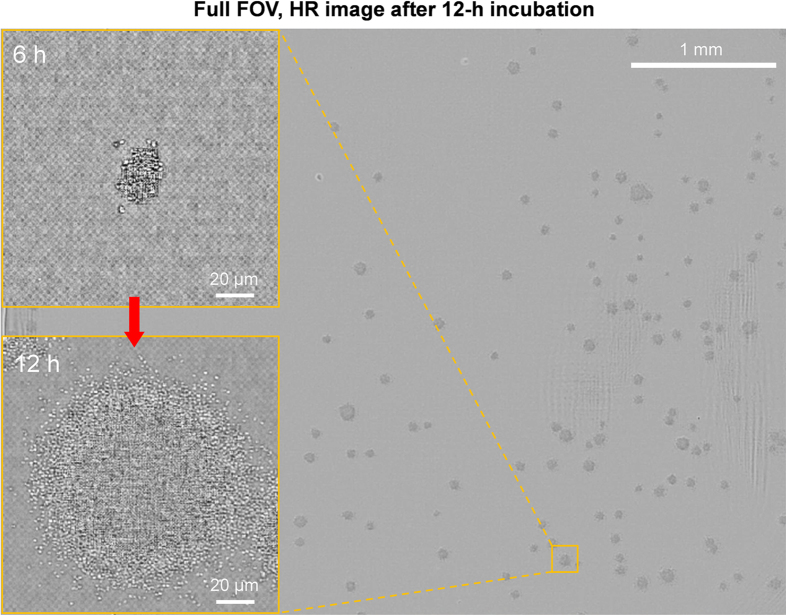
Full field-of-view (5.7 mm × 4.3 mm) of S. epidermidis microcolonies after incubation for 12 h. The insets show the variation in colony size with the incubation period at the same location.

Figure 2 shows a representative, reconstructed HR image of bacterial colonies with full FOV (5.7 mm × 4.3 mm) following 12-h incubation period. The ePetri platform collected microscopy images over the entire area of the sensor, providing a wide FOV that was several orders of magnitude larger than the FOV of a conventional microscope but with comparable resolution. Additionally, the magnified insets of Fig. 2 show a comparison between images of a single bacterial colony after 6- and 12-h incubation periods at same location. This shows that our system can track individual bacterial colonies from a very early stage and, hence, can provide spatiotemporal distributions during colony growth. In the pixel super-resolution reconstruction process, the SPSM method allows for digital focusing of the images^29^. In this study, we used the depth of the imaging plane which resolved the boundary of bacterial colony (further details can be found in Fig. S2). Additionally, single bacterial colony movies are provided in the Supplemental Information (see Movie S1). We observed the merging of neighboring bacterial microcolonies, which has implications for colony counting using conventional methods. In the conventional bacterial colony-counting protocol, a colony density of <10 CFU/cm^2^ is recommended to avoid the merger of neighboring colonies^43^. If we count a 20-μm-diameter bacterial microcolony, the recommended colony density would be <25,000 CFU/cm^2^, which is approximately proportional to conventional colony density (i.e., assuming that a 1-mm-diameter colony is detectable on conventional colony counting). In all experiments, we set a microcolony concentration of <100 CFU/cm^2^ on the CMOS image sensor.

### Microcolony counting

To automatically detect microcolonies from the time-lapse HR image sequences, a simple edge-detection image-processing algorithm was implanted in MATLAB and used. This algorithm consists of three steps: 1) image loading, 2) segmentation of colonies, and 3) enumeration and sizing of colonies. Details of the image-processing algorithm are described in Fig. S3.

**Figure 3:**
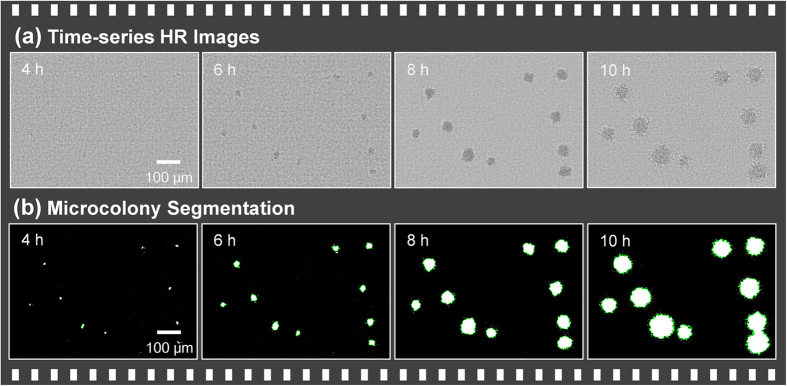
Image processing. (**a**) Time-series high-resolution (HR) images of bacterial microcolonies reconstructed using sub-pixel sweeping perspective microscopy (SPSM). (**b**) Following reconstruction of the HR image, bacterial microcolonies are segmented using a custom MATLAB program (see Fig. S3). The equivalent diameter and number of colonies in each time-lapse image are then calculated.

For real-time bacterial microcolony recognition, the ePetri system automatically captures a sequence of bacterial cell growth events across the entire sensor chip, which is used to track the growth pattern of bacterial microcolonies. Figure 3(a) shows time-series HR images of growing bacterial colonies, Fig. 3(b) shows corresponding processed images, which were used to detect colonies. Based on these time-lapse imaging data, we were able to detect and track individual bacterial colonies in both space and time. The exterior boundaries of each of the detected microcolonies was identified according to the growth rate and used to measure the area of the colony, which was then converted into the diameter of a circle with the same area. Based on these data, the size and concentration of colonies could be characterized in real time. Dynamic results of this process are shown in Movie S2.

**Figure 4:**
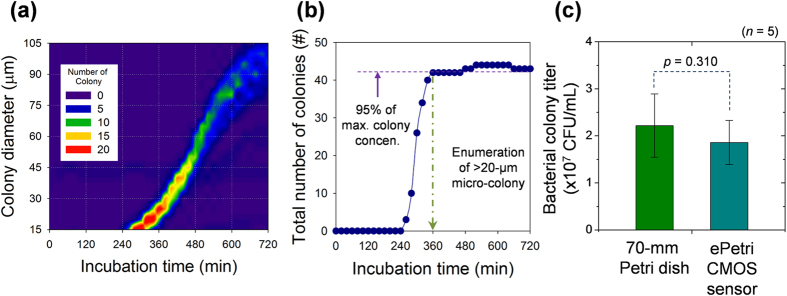
Real-time measurements of S. epidermidis microcolonies. (**a**) The bacterial microcolony size distribution as a function of the incubation time. The time interval for image acquisition and processing was 20 min. The bacterial microcolony size distribution with different incubation periods. Their average diameter and standard deviation were 24 ± 5.9 μm after 360 min, 51 ± 9.9 μm after 480 min, 79 ± 18.5 μm after 600 min, and 93 ± 23.1 μm after 700 min. (**b**) The number of colonies with a diameter of ≥20 μm as a function time. The number of colonies started to increase after 240 min and saturated to 95% of the maximum after 360 min. (**c**) The performance of the ePetri microcolony assay and of conventional colony counting. The average colony titer given by the ePetri microcolony assay was 1.9 × 10^8^ CFU/mL, which is 13% less than that given by the conventional colony-counting assay (i.e., 2.2 × 10^8^ CFU/mL). However, the difference in performance of the two methods was not statistically significant (*p* = 0.310, *n* = 5; Mann–Whitney U-test).

As a demonstration, we monitored the growth of *S. epidermidis* bacterial microcolonies. Figure 4(a) shows the size distribution of a bacterial colony as a function of the incubation time. The equivalent colony diameter increased monotonically with the incubation time. The number of bacterial microcolonies that were >20 μm in diameter saturated after 360 min to ~95% of the maximum, as shown in Fig. 4(b). The number of bacterial colonies increased rapidly as a function of the incubation time once the size had reached the threshold for detection, and the cell count reached 50.1 ± 1.67 CFU after 12 h. The incubation time to reach saturation of the total number of colonies may be reduced further by improving the clarity of the image acquisition process and reducing noise by using a more advanced image-processing algorithm for cell segmentation. Furthermore, a smaller initial colony size for colony enumeration leads to faster saturation of the colony number (see Fig. S4). In this study, we considered >20-μm-diameter bacterial microcolonies for the reliable enumeration.

### Comparison with conventional colony counting

To compare the performance of our ePetri microcolony assay with that of a conventional colony-counting assay, we grew bacterial colonies on 70-mm-diameter Petri dishes and on the CMOS image sensors. For the measurement of initial titer of the *S. epidermidis* suspension, the bacterial suspension was serially diluted and plated onto the nutrient agar according to the conventional colony-counting assay. Then, the cultured colonies were counted using the naked eye after incubation for 24 h at 37 °C. The bacterial colony titer given by the conventional colony-counting assay was 2.2 ± 0.67 × 107 CFU/mL (Average ± Standard deviation). The same *S. epidermidis* suspension sample was used for the ePetri microcolony assay with 1:10^2^ dilution. For the ePetri group, the HR images were acquired at 20-min intervals up to 12 h after incubation, and our microcolony recognition algorithm was used to automatically count the number of microcolonies (see Fig. 4(c)). The bacterial colony titer given by the ePetri microcolony assay was 1.9 ± 0.47 × 107 CFU/mL. According to a Mann-Whitney U-test, there were no significant differences between both methods (*p* = 0.310, *n* = 5).

In this study, we tested only one bacterial species as a simple comparison of the ePetri microcolony assay with conventional colony counting. The test results showed that the performance of the ePetri CMOS sensor platform was close to that of conventional colony counting. It is worth noting that further comparison studies using various bacterial species and environmental conditions are necessary to demonstrate equality with the current gold standard. In addition, although we set the microcolony concentration of <100 CFU/cm^2^ on the CMOS image sensor, further research about the concentration range that can be dealt with using the ePetri platform is necessary.

## Discussion

We demonstrated real-time bacterial microcolony counting using the ePetri on-chip microscopy platform.

This platform has several major advantages for microcolony counting in comparison with conventional methods. First, the imaging area of the sensor measures ~25 mm^2^, whereas the FOV of a 20× objective lens (e.g., Olympus, Plan N, NA = 0.4) measures approximately 1 mm^2^. The wide FOV of the ePetri platform supports the simultaneous observations of multiple microcolonies. Second, the SPSM super-resolution algorithm provides sub-micron resolution, which enables earlier identification of microcolony sites than is possible with conventional colony-counting assays using the naked eye. Note the short readout time of 6 h, which is due to the ability of the ePetri microscopy platform to recognize *S. epidermidis* microcolonies from their early stages (i.e., when the diameter of the bacterial microcolony is ≥20 μm). Third, the automated real-time imaging process is continuous during the cell incubation period, which not only saves labor, but also allows the stable monitoring of colony growth without disturbing the sample. The ePetri microscopy platform has significant potential as a low-cost portable solution for the microscopy-based diagnosis of various microorganisms because of its compact size (10 cm × 10 cm × 10 cm) and simple configuration of mass-producible electronic components without optical lenses.

Previous studies demonstrated that the SPSM method allows for digital refocusing of the images^29^,^44^. In the pixel super-resolution reconstruction process, the low-resolution frames are arranged with the specific shift corresponding to the depth of the imaging plane and reconstructed to high-resolution images at multiple depths without tuning the focus during image acquisition. When we set the focal plane for the super-resolution reconstruction process to the depth of a proximate single bacterial cell, we could obtain a clear colony boundary and increased the speed of the reconstruction process while the image of center region of the colony, which was thicker than the boundary, was out of the focal plane. Also, if the samples move farther away from the sensor, the imaging resolution will deteriorate due to the diffraction of the shadows.

In this study, a simple edge-detection image-processing algorithm was used for microcolony segmentation and sizing. The future development of an advanced cell-recognition algorithm is able to detect newly generated colonies, track the growth of each individual colony, and distinguish different colonies after they contact one another, is required to provide more valuable information (e.g., regarding the frequency of colony mergers, which lead to the underestimation of microcolonies^37^,^45^,^46^).

We believe that this real-time microcolony-counting technique would be valuable as a rapid diagnostic platform for the detection of pathogens or diseases, the study of microorganisms, and the discovery of new antimicrobial drugs as well as a general tool for biomedical and bioscience research. In particular, the ability to monitor the colony-formation dynamics in real-time presents the possibility for further use in clinical microbiology and microbial research.

## Method

### Preparation of test bacteria

*S. epidermidis* (ATCC 12228) was used as the test bacteria. This gram-positive bacterial strain is commonly used in bioaerosol research^47^,^48^. *S*. epidermidis is currently seen as important opportunistic pathogen, but their infections only rarely develop into life-threatening diseases. In particular, *S. epidermidis* represents the most common source of infections on indwelling medical devices^49^. Bacterial cultures were grown in tryptic soy broth (TSB; Becton Dickinson, Franklin Lakes, NJ) at 37 °C for 18 h.

### Preparation of nutrient agarose coverslip

Coverslips were prepared using 1.5% agarose with nutrient broth (Becton Dickinson) in 70-mm-diameter Petri dishes. Using a Harris Uni-core biopsy punch (Ted Pella Inc., Redding, CA, USA), the coverslip (4.0 mm in diameter and 1-mm thick) was loaded onto the CMOS sensor.

### Preparation of CMOS image sensor chip

We used an MT9P031 image sensor (2.2-μm pixel, Aptina)^50^. The glass cover of each sensor was removed by cutting the edges of the glass on a hot plate (180 °C). The microlens layer was removed using oxygen plasma (10 min at 120 W) to provide direct access to the sensor pixels. After the experiments, the image sensors and PDMS covers were reused following sterilization by the autoclave (120 °C for 15 min) and cleaning with deionized water in an ultrasonic bath for 30 s at 70 °C.

## Additional Information

**How to cite this article**: Jung, J. H. and Lee, J. E. Real-time bacterial microcolony counting using on-chip microscopy. *Sci. Rep*. **6**, 21473; doi: 10.1038/srep21473 (2016).

## Supplementary Material

Supplementary Information

Supplementary Movie S1

Supplementary Movie S2
