# Supplementary material for: Real-time bacterial microcolony counting using on-chip microscopy
Source: Sci Rep. 2016 Feb 23;6:21473. doi: 10.1038/srep21473 (PMC4763285; doi:10.1038/srep21473)
Supplement: Supplementary Information [file srep21473-s1.doc]

**Supplementary Information for**

**“Real-time bacterial microcolony counting using on-chip microscopy”**

^a,b^Jae Hee Jung* and ^c^Jung Eun Lee

^a^Electrical Engineering, California Institute of Technology, Pasadena, CA 91125, USA

^b^Center for Environment, Health, and Welfare Research, Korea Institute of Science and Technology, Seoul 136-791, Republic of Korea

^c^Han-River Environment Research Center, National Institute of Environmental Research (NIER), Yangseo-myeon, Yangpyeong-gun, Gyeonggi-do 476-823, Republic of Korea

*Correspondence should be addressed to: [jaehee@kist.re.kr](mailto:jaehee@kist.re.kr); Tel.: 82-2-958-5718

**Figure S1:**

**Fig. S1. Schematic diagrams of the sub-pixel sweeping perspective microscopy (SPSM) technique for the ePetri platform.** Each LED in the 8 × 8 LED array is turned on sequentially. By sweeping the LED illumination using an incremental tilt/shift process (Fig. S1 (a)), the image sensor captures the light transmitted through the sample under each LED. A sequence of bright-field low-resolution (LR) images (limited by the pixel size of 2.2 μm) is then obtained (Fig. S1 (b)). Each frame represents a view of the object that is laterally shifted with respect to the next due to the varying angle of incidence of the illumination. A shift-and-add pixel superresolution algorithm is applied to construct an HR image. This algorithm shifts each LR image by the relative sub-pixel shift given by the computed illumination position vector (Fig. S1 (c)). These are then summed to fill a blank HR image grid, with an enhancement factor *n*, where the *n* × *n* pixel area of the HR image grid corresponds to a single pixel area of the LR image grid (Fig. S1 (d)). In brief, the superresolution construction accounts for the different spatial information obtained in each of the 64 LR images and creates a single HR image. Further details of the image acquisition and construction algorithm have been described by us previously.

**Figure S2:**

**Fig. S2. Time-series images of single bacterial microcolony acquired using the ePetri platform.** (a) The target object rests on the surface of the image sensor, and sequential low-resolution (LR) images of the bacterial cell are captured using the sensor with the moving illumination source. (b) Each LR sequence is used to construct a single monochromatic high-resolution (HR) image using the superresolution algorithm. From this HR image, the boundary of the bacterial colony was clearly resolved, which can be seen starting as a single cell.

In the pixel superresolution reconstruction process, the SPSM method allows for digital focusing of the images.[^2^](#_ENREF_2) The LR image frames are arranged with the specific shift corresponding to the depth of the imaging plane. The same set of raw data can be reconstructed to a HR image at multiple depths. Therefore, the bacterial microcolonies with variable dimensions could be imaged without tuning the focus during image acquisition. In this study, we used the depth of the imaging plane which resolved the boundary of bacterial colony. On the contrary, the middle of the colony, which is thicker than the colony boundary, cannot be resolved clearly because of the different focal image plane (see Fig. S2 (b) – 6 h).

**Figure S3:**

**Fig. S3. Bacterial colony segmentation using a simple edge-detection image-processing algorithm.** The edge-detection image-processing algorithm was implemented using MATLAB[^3^](#_ENREF_3) and consists of three steps, which are described as follows:

**1. Image loading.** A new frame from the time-series HR image sequence is loaded into the program (see Fig. S3 (a))

**2. Colony segmentation.** A brightness threshold is set for the image to convert the HR image into a binary mask that contains the segmented colony (see Fig. S3 (b)). In the binary gradient mask, linear gaps in the lines of high contrast surrounding the colony object are eliminated using linear structuring elements (see Fig. S3 (c)), and the residual holes in the interior of the colony object are filled (see Fig. S3 (d)). To make the segmented object appear more natural, the object was smoothed by eroding the image twice using diamond structuring elements (see Fig. S3 (e)).

**3. Enumeration and colony sizing.** Using the exterior boundaries of objects in the image (see the green outlines in Fig. S3 (f)), the area of each object is measured, and the diameter of an equivalent circle with the same area is calculated. The size and concentration of colonies in each time-series image is then obtained in real time.

**Figure S4:**

**Fig. S4. Saturation of the number of microcolonies.** Figure S4 (a) shows the total number of microcolonies as a function of the minimum size in the colony counting algorithm and the incubation period. Figure S4 (b) shows a subset of these data. Using the bacterial segmentation image-processing algorithm, the number of bacterial colonies greater than a given size was counted. With a countable minimum size of <20 µm, some counting error occurred during image processing. However, with a minimum size of 20 µm, the initial colony detection time was 300 min and the time to reach 95% of the maximum colony concentration was 360 min.

**Movie S1:**

**Mov. S1. Time-series images of live bacterial colonies.** Movie S1 shows high-resolution (HR) time-series images of bacterial colonies from two specific locations. The left movie shows an initial bacterial cell growing to form a bacterial colony. The right movie shows initial bacterial cells separated by ~6 μm. After 4 h, these merged form for a single colony. Such closely neighboring bacterial microcolonies may be considered to result in an underestimate of the number of colonies with conventional bacterial colony counting. Using the ePetri microscopic colony counting system, we could enumerate single bacterial microcolonies before they merged.

**Movie S2:**

**Mov. S2. Real-time image processing of time-series high-resolution (HR) images of live bacterial microcolonies.** At each time interval, sequential low-resolution (LR) raw images of bacterial cells on the surface of the image sensor were captured using the sensor, with a moving illumination LED source. These were used to construct an HR image using the superresolution algorithm. Following this process, bacterial microcolonies were segmented using the custom MATLAB program, as described in Figure S3. The diameter of an equivalent circle with the same area of the microcolony was then calculated, and the number of colonies in each image was counted.

**References**
